# Comparison of analyses of the QTLMAS XIII common dataset. II: QTL analysis

**DOI:** 10.1186/1753-6561-4-s1-s2

**Published:** 2010-03-31

**Authors:** Chris Maliepaard, John W M Bastiaansen, Mario P L Calus, Albart Coster, Marco C A M Bink

**Affiliations:** 1 Plant Breeding, Wageningen University, Wageningen, The Netherlands; 2 Animal Breeding and Genomics Centre, Wageningen University, Wageningen, The Netherlands; 3 Animal Breeding and Genomics Centre, Wageningen UR Livestock Research, Lelystad, The Netherlands; 4 Biometris, Wageningen UR, Wageningen, The Netherlands

## Abstract

**Background:**

Five participants of the QTL-MAS 2009 workshop applied QTL analyses to the workshop common data set which contained a time-related trait: cumulative yield. Underlying the trait were 18 QTLs for three parameters of a logistic growth curve that was used for simulating the trait.

**Methods:**

Different statistical models and methods were employed to detect QTLs and estimate position and effect sizes of QTLs. Here we compare the results with respect to the numbers of QTLs detected, estimated positions and percentage explained variance. Furthermore, limiting factors in the QTL detection are evaluated.

**Results:**

All QTLs for the asymptote and the scaling factor of the logistic curve were detected by at least one of the participants. Only one out of six of the QTLs for the inflection point was detected. None of the QTLs were detected by all participants. Dominant, epistatic and imprinted QTLs were reported while only additive QTLs were simulated. The power to map QTLs for the inflection point increased when more time points were added.

**Conclusions:**

For the detection of QTLs related to the asymptote and the scaling factor, there were no strong differences between the methods used here. Also, it did not matter much whether the time course data were analyzed per single time point or whether parameters of a growth curve were first estimated and then analyzed.

In contrast, the power for detection of QTLs for the inflection point was very low and the frequency of time points appeared to be a limiting factor. This can be explained by a low accuracy in estimating the inflection point from a limited time range and a limited number of time points, and by the low correlation between the simulated values for this parameter and the phenotypic data available for the individual time points.

## Background

The abundant availability of DNA markers in many plant and animal species allows geneticists and breeders to identify and quantify the contributions of genomic regions to quantitative traits. The genetic inheritance of agriculturally important quantitative traits is often complex and frequently interacts with temporal and spatial (environmental) variation.

Time-dependent traits may be analysed in different ways. The most straightforward approach is to consider the trait at each time point separately by performing independent QTL analyses at each single time point. Subsequently, the results of these analyses are summarized and interpreted. A disadvantage of this approach is that it does not explicitly consider the correlations between observations at the individual time points. In the interpretation this may lead to speculations about QTLs that appear and disappear in certain phases of the life cycle, while they may in fact be QTLs influencing developmental processes rather than the trait at a certain time point. An alternative approach is a two-step approach where in the first step for each individual in the population a curve is fitted over the time points, *e.g.* a logistic or Gompertz growth curve. In the second step the estimated parameters of the curves or derived estimates are considered as the quantitative traits in a QTL analysis. For example, these parameters might be the derivative in the exponential part of the curve, area under the curve, time to reaching the inflection point. In this way the data of the whole time series is considered simultaneously and a biological interpretation can be given to identified QTLs: for example, QTLs might influence growth rate or earliness or maximum size of the animals or crop. A third alternative approach is to employ a single-step approach in which the covariance structure between the time points is allowed for and the effects are estimated simultaneously with additional fixed and random terms such as polygenic effects and environmental factors. All these approaches have been applied successfully in QTL mapping of time-dependent traits [1-8]. The aim of this study was to evaluate methods used by participants of the QTL- MAS 2009 workshop to detect QTLs related to time course data of a common data set.

## Methods

### Simulated data

**Figure 1:**
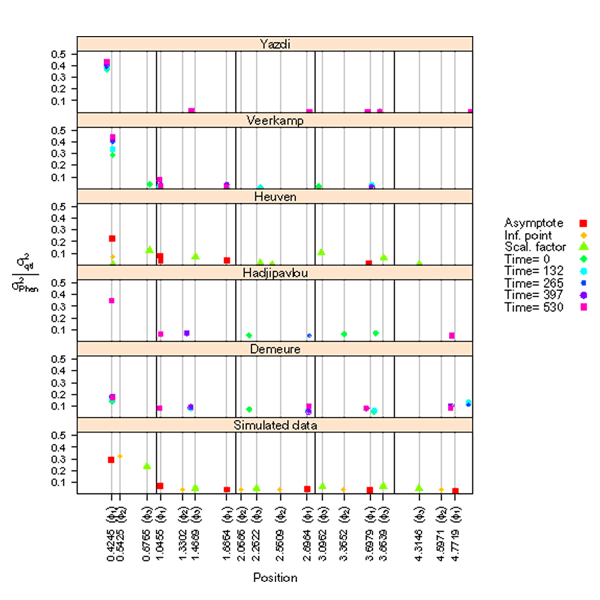
**Map positions of simulated and reported QTLs and their percentage explained phenotypic variances.** Black lines separate the five chromosomes. Grey lines and x-axis labels indicate the map positions of six simulated QTLs per parameter of the growth curve. QTL may pertain to the three curve parameters, i.e., Asymptote (Φ_1)_ - inflection point (Φ_2_) - scaling factor (Φ_3_) or the trait at five time points. Only QTLs reported to have additive effects are shown.

Details of the simulation are described separately [9]. The dataset presented to participants of QTLMAS XIII consisted of 100 full-sib families which resulted from factorial mating of 20 female and five male parents. Each full-sib family consisted of 20 offspring. Parent-offspring relationships were provided, but relationships between parents were not given. Genotype data of 453 diallelic SNP markers distributed over five chromosomes of one Morgan each, were available for parents and offspring. Phenotypes were available for the offspring of 50 full-sib families and consisted of cumulative yield records measured at five distinct points in time, the last time point being 530. In total, 18 additive QTLs were simulated, six affecting each of three parameters of the logistic growth curve (Table 1, Figure 1). These parameters were the upper asymptote (φ_1_), the inflection point (φ_2_) and the scaling factor (φ_3_). For each parameter, the simulated heritability over the six QTLs was 50%, divided over one large QTL (23-32% of phenotypic variance) and five small QTLs (2.5-7.1%). The large QTLs were simulated on chromosome 1, the small ones were distributed over the other five chromosomes. Participants were requested to perform a QTL analysis and to report estimated QTL positions and explained variance of each QTL.

**Table 1:** Simulated QTLs: chromosome, positions, parameter name, effect sizes in percentage explained phenotypic variance, percentage of informative full-sib families for the QTL (percentage of crosses with at least one heterozygous parent), the maximum amount of LD (R2) with any marker, and the number of participants who detected the QTL.

Chrom	QTL pos	Parameter	Effect size QTL	%Inf. Families	MaxLD	Times found
1	42.45	Asymptote	29.3%	40	0.96	4
2	4.55	Asymptote	7.1%	68	0.57	4
2	88.64	Asymptote	3.7%	64	0.73	2
3	89.94	Asymptote	4.1%	58	0.38	4
4	69.97	Asymptote	3.3%	78	0.80	4
5	77.19	Asymptote	2.5%	82	0.16	1
1	54.25	Infl. Point	32.3%	62	0.24	0
2	33.02	Infl. Point	3.5%	48	0.89	0
3	6.86	Infl. Point	3.5%	34	1.00	0
3	56.09	Infl. Point	3.8%	70	0.26	0
4	36.52	Infl. Point	3.2%	84	0.35	1
5	59.71	Infl. Point	3.7%	80	0.55	0
1	87.65	Scaling factor	23.4%	50	0.95	2
2	48.89	Scaling factor	4.8%	64	0.99	2
3	26.22	Scaling factor	4.7%	80	0.44	3
4	9.62	Scaling factor	5.9%	34	0.54	3
4	86.39	Scaling factor	6.6%	74	0.80	2
5	31.48	Scaling factor	4.6%	68	0.21	1

### Post-workshop data set expansions

In order to find out what the most important limitations were in the QTL analysis, we expanded the common data set which was available to the participants, to a longer time frame (up to 1010 instead of 530) and more time intervals (intervals of 20 instead of about 130). We investigated four data sets:

W1: the common data set: 5 time points from 0 up to 530, time intervals of about 130.

W2: expanded data set: 9 time points from 0 to 1010, time intervals of about 130.

W3: expanded data set: 27 time points from 0 to 530, time intervals of 20.

W4: expanded data set: 51 time points from 0 to 1010, time intervals of 20.

Only data set W1 was available to the participants. We use W2 to W4 to explore possible limitations in W1 for QTL analysis with regard to the number of time points available and the size of the time intervals (Additional file 1).

### Estimation of growth curve parameters and their correlations

For the common data set, genotypic values for the parameters of the growth curve were first transformed into phenotypic values (environmental noise added) and then cumulative yield of the individuals at the time points was calculated from those parameter values, as detailed in [9]. Therefore, even if the correct model is used to estimate the parameters of the growth curve, there are two limitations in the accuracy of these estimates: the quality of the parameter estimation based on a limited set of time points (five) in a limited time frame (only up to time point 530), and, in addition, the heritability: how well does the phenotype reflect the true genotypic values. In order to evaluate the importance of each of those two limitations we calculated correlations between genotypic and phenotypic values of the parameters, and correlations with the parameters estimated from the common data set and the three expanded data sets as listed above.

### Estimated variances explained by the QTLs

Participants reported QTL effect size either in units of the phenotype or in terms of explained phenotypic variance. To allow proper comparison we transformed all reported estimates into proportion explained phenotypic variance. In two studies by participants Heuven & Janss [10] and Veerkamp et al. [11], QTL effect sizes were initially reported and the corresponding additive genetic variances were calculated from [12], where *a* is the allele substitution effect and *p* denotes the allele frequency. Furthermore, [10] provided estimates of the total phenotypic variances in their Table 2 for the three curve parameters and these were used here to calculate the explained variance of the QTLs reported. [11] did not provide phenotypic variance estimates and the values 0.10, 0.67, 3.76, 15.56, and 39.17 were taken as estimates for the phenotypic variances at the five time points.

**Table 2:** Numbers of reported and correctly identified QTLs.

**Participant**	**# Reported**	**# Correct**	**# Asymptote**	**# Inflection point**	**# Scaling factor**
					
Demeure et al. [12]	9	4 (8)	4	0	0
Hadjipavlou et al. [13]	15	8 (13)	4	1	3
Heuven & Janss [10]	9	8 (9)	4	0	4
Veerkamp et al. [11]	14	9 (14)	5	0	4
Yazdi et al.	6	4 (5)	2	0	2

Numbers between parentheses indicate the numbers if reported QTLs were within 10 cM (instead of 5 cM) of a simulated QTL and if more than one reported QTL within that interval are all considered correct.

### Methods used by the participants

Most participants used more than one method for the detection of QTLs; additionally, in most cases the yield at the time points were taken as the phenotype to be analyzed, in other cases parameters of a logistic or Gompertz growth curve, or the growth between two time points. Participants usually had a clear preference for one of the methods they used and only results of this preferred method are included here for the main comparison of QTL analysis results.

Demeure et al. [12] used linkage analysis interval mapping (LA-IM) for QTL detection. Hadjipavlou et al. [13] and Yazdi et al. [personal communication] used a variance component model including estimated IBD probabilities. Heuven & Janss [10] and Veerkamp et al. [11] both used a Bayesian variable selection method following [14], also known as BayesC, where two prior distributions are used to allow for SNPs with a large effect and SNPs with a small effect. Heuven & Janss [10] assumed a fixed proportion of 30% of the SNPs to have a large effect, while Veerkamp et al. [11] estimated this proportion from the data.

Four of the five participants analyzed the yield at the five time points as independent observations. Heuven & Janss [10] estimated parameters of a logistic growth curve, which was the same model as used to generate the data, and performed QTL analysis on these parameters. Hadjipavlou et al. [13], in addition to analyzing single time points and growth between time points, also analyzed the estimated parameters of a Gompertz curve but the estimates of the Gompertz curve were not considered in the variance component method presented here.

### Comparison

QTLs were considered as true positive results if the reported QTL was within 5 cM of a simulated QTL. If two QTLs were reported within 5 cM of a simulated QTL, only one was declared as a true positive and the shortest distance to the QTL was taken into account for calculation of the position accuracy. Consequently, the second QTL was declared as a false positive.

In a second comparison we also counted as true positives the QTLs within 10 cM of a simulated QTL and we allowed multiple reported QTLs within that same distance (a second QTL was not declared as false positive).

## Results

### Estimation of the growth curve parameters

QTLs affecting the three growth parameters were simulated on each of the five chromosomes (Table 1. Figure 1), but only cumulative yield at five time points was available to the participants. The limitations in estimating these parameters from the cumulative yield at a number of time points are different for the three parameters of the growth curve: for the asymptote (φ_1_) and the scaling factor (φ_3_), the limiting factor is mostly the heritability: correlations between the estimated parameters and the phenotypic values are very high (> 0.98 for φ_1_, > 0.90 for φ_3_), but the correlation between phenotypic values and genotypic values is only about 0.7 (corresponding to a heritability of 50%). For the asymptote (φ_1_) increasing the time range to 1010 or shortening the time intervals to 20 does not make much difference: the five time points are already sufficient to estimate the phenotypic asymptote accurately. For the scaling factor (φ_3_), increasing the time range and intervals helps marginally, as the main limiting factor is still the low correlation between phenotypic and genotypic values. However, for the inflection point (φ_2_) both number of time points and the range of the time are limiting: if the parameters are estimated over a time range up to time point 1010 (instead of 530) and intervals of 20 are provided, the correlation with the phenotypic φ_2_ is 0.98, but with a smaller time range and a smaller number of intervals this decreases to 0.65. The correlation between the genotypic φ_2_ and the estimated φ_2_, is only 0.48. So, for the inflection point both the heritability and the limitations in the number and range of the time points are decreasing the accuracy of estimating the parameter, and therefore the power to detect the underlying QTLs.

### Number of QTLs detected

The number of QTLs reported by the participants varied from 6 to 15 (Table 2). Over the five participants, in 13 cases a significant effect was reported within 5 cM of a simulated QTL, mostly for QTLs affecting the asymptote and the scaling factor. In only one case, Hadjipavlou et al. [13], a QTL was reported within 5 cM of a QTL affecting the inflection point. All QTLs for the asymptote and the scaling factor were discovered by at least one of the participants (Table 2). None of the 18 QTls was correctly reported by all the participants, but three of the QTLs for the asymptote were reported by four participants each. Demeure et al. [12] and Yazdi et al. [personal communication] each detected four QTLs correctly, Heuven & Janss [10] and Hadjipavlou et al. [13] detected eight, and Veerkamp et al. [11] detected nine QTLs. In some cases, notably by Hadjipavlou et al. [13] and Veerkamp et al. [11], the effect of a single QTL was split over more than one reported significant SNP near the QTL; in some other cases a reported significant effect was just outside the 5 cM region but still within 10 cM distance. If these situations are also considered the number of detected QTLs is higher (Table 2, numbers between parentheses). Under those criteria the number of false positives is reduced to zero to two for all participants. However, it should be noted that a 10 cM interval on both sides of each of 18 QTL positions would cover a large proportion of the simulated genome. That is, 333 out of 500 cM would be considered 'correct', so a probability of about 2/3 in the case of random assignment of the correct number of QTLs.

### Estimated positions of the QTLs

Simulated and reported positions of QTLs are shown in Figure 1. For those authors that used a method that tested individual SNP positions, reported SNPs were usually in medium to high LD with the nearest QTL. For example, the 14 SNPs reported by Veerkamp et al. [11] had LD values (R2) with the QTL ranging from 0.16 to 0.96 (our calculations, results not shown). However, from the nine SNPs reported by Heuven & Janss [10], all nine from analyses using single SNP haplotypes, three had an R2 value smaller than 0.10 with the nearest simulated QTL. Estimated average distances over the reported true positive QTLs were smallest for Hadjipavlou et al. [13] (1.7 cM, Table 3) and largest for Yazdi et al. (3.9 cM). Over all reported QTLs, including the ones reported outside the 5 cM interval and including multiple reported QTLs within the 5 cM interval, the average distance was smallest for Heuven & Janss [10] (2.0 cM) and highest for Yazdi et al. (6.8 cM, Table 3).

**Table 3:** Distances of reported QTL positions to simulated QTL positions


**Participant**	**True positives**	**All**	
Demeure et al. [12]	2.2 cM	5.7 cM
Hadjipavlou et al. [13]	1.7 cM	4.9 cM
Heuven & Janss [10]	1.9 cM	2.0 cM
Veerkamp et al. [11]	2.3 cM	2.7 cM
Yazdi et al.	3.9 cM	6.8 cM

### Estimated variances explained by the QTLs

The reported variances of the QTLs were consistent with the simulated values, *i.e.,* largest variances were reported for QTLs on chromosome 1 and reported variances on other chromosomes were relatively small. However, overestimation of QTL variances on chromosome 1 occurred in the studies by Yazdi et al. and by Veerkamp et al. [11], while estimated variances for QTLs on chromosome 1 reported by Demeure et al. [12] and also Heuven & Janss [10] were below the simulated effect sizes. Demeure et al. [12] reported relatively high estimates for variances of QTLs on the other chromosomes.

### Dominance, epistasis, imprinting, multiple QTL tests

Only additive QTLs were simulated but some participants reported dominance, imprinting or epistatic effects. Hadjipavlou et al. [13]mentioned explicity that *no* epistatic QTLs were detected. However, they did report dominant and imprinted QTLs. Demeure et al. [12] reported epistatic QTLs but these are false positive results. Tests for multiple QTLs sometimes resulted in false positive results, for example in the case of Demeure et al [12], who performed a test for multiple QTLs and reported two QTLs at chromosome 3 at 17 cM and 48.7 cM. In the methods used by Hadjipavlou et al. [13] and Veerkamp et al. [11] the effect of a single QTL was sometimes split up over two SNPs near the simulated position. Heuven & Janss [10] applied two additional analyses to retrieve situations in which a group of markers might be associated with a QTL. However, this still led to situations where two QTLs were detected while in fact only one was present. They indicated that their window- based approach to find evidence for multiple QTLs was sometimes in contradiction with individual values of their 'parameter-wise' Bayes Factor per marker.

## Discussion

**Figure 2:**
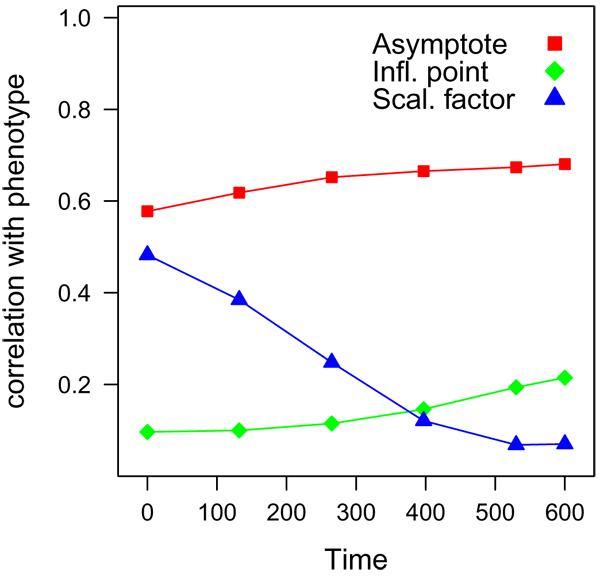
Correlation of the true values for the parameters of the logistic growth curve with cumulative yield at the five time points (0, 132, 265, 397, 530)

QTL detection was most powerful for the QTLs affecting the asymptote of the logistic growth curve. This can be explained by the fact that the correlation between yield and the asymptote was higher than correlation between yield and the other two parameters of the growth curve, especially at the later time points (Figure 2). There was also a relatively high correlation (0.69) between the true values of the asymptote and the asymptote values estimated from yield at the five time points (if a logistic growth curve is used to estimate the parameters). In agreement with this, some authors mentioned explicitly that the association was clearest for the later time points. Each QTL for the scaling factor was also reported by at least one of the participants.

Correlation between the scaling factor and yield was highest for the early time points, but always below the correlation of the asymptote with yield (Figure 2). The correlation between true values for this parameter and the values estimated from the logistic growth curve was 0.67. Some authors observed that these QTLs were usually just found for early time points (0 or 132) and significance of these QTLs decreased over the time points. Only one reported QTL was near a simulated QTL for the inflection point. Correlation between yield and the simulated values for this parameter was low in all time points (< 0.20; Figure 2) and also the correlation between true and estimated parameter values is smaller for the inflection point (0.48) than for the asymptote and the scaling factor. Here not only the heritability is limiting but also the estimation of the parameter from the limited time range and small set of time points available. The impact of the frequency and range of time points available was explored in more detail by analyses of extensions of the original common dataset using FlexQTL software [15,16]. See Additional file 1 for the details on these analyses.

The QTL for the asymptote on chromosome 5 was reported by only one of the participants. A possible explanation is given by the small effect of this QTL (4.6% of phenotypic variance) and the low level of LD between this QTL and the markers. The maximum LD measured as R^2^ was 0.16 for a marker at 14 cM distance from this QTL.

The number of informative crosses for a QTL (where at least one of the parents is heterozygous for the QTL alleles) did not seem a very limiting factor in the QTL analysis even though it varied between 34% and 84% (Table 1).

The Bayesian methods of Heuven and Janss [10] and Veerkamp et al. [11] and the variance component method as used by Hadjipavlou et al. [13] were the most successful for identifying the QTLs in this data set. Whether or not the correct growth model was used did not matter much for the QTL detection. Participants who analysed yield at the individual time points, or parameters from a Gompertz curve also reported similar results for this QTL analysis.

There is a large number of false positive results when we consider as true positives the reported QTLs within 5 cM of the simulated position and counting only a single true positive within the resulting 10 cM bracket. However, under the milder criterion of a 20 cM bracket (10 cM distance from a simulated QTL) and counting multiple reported QTLs within the bracket as true positives, the numbers of true positives are much larger and there are only 0 to 2 false positives for all participants (numbers between parentheses in table 2).

The simulated QTLs had only additive effects. No dominance effects, epistasis or imprinting were present. Still these effects were reported by participants. Detection of imprinted QTLs can possibly be explained by varying numbers of fathers / mothers that are heterozygous for markers and QTLs. The reported evidence of dominant, epistatic and imprinted QTLs where none were simulated suggests that the control of type I errors in these non-additive models needs to be considered more carefully. Also the methods used by participants to distinguish between a single QTL and multiple linked QTL within a window of markers did not seem very successful. This may also indicate that better control of false positives is also needed in the case of multiple QTL models.

## Conclusions

QTL analysis of time-related traits can be applied successfully with a range of methods as shown by the participants of the QTL-MAS workshop 2009. The power to detect QTLs for growth related traits in this study depended critically on the size of the variances, the amount of LD with nearby markers and on the accuracy with which parameters could be estimated from the time point data and on the correlation between time point data and the actual genotypic values for growth characteristics. This simulated data set was very useful in showing the power and limitations to estimate QTLs in time-related traits in situations where the QTLs are involved not so much in the expression of the trait at a single time point but influencing the characteristics of development. For future simulated data sets we would recommend to include also chromosomes with no QTLs and with QTLs showing no linkage disequilibrium with any of the marker haplotypes to better establish false positive rates. Results of the QTL-MAS 2009 workshop also indicate that special attention is needed for controlling false positive results for non-additive models and multiple QTL models. All participants who performed QTL analysis on the common data set were animal breeders while the dataset may be representative for plant breeders as well. We hope that in future workshops the choice of the challenge and properties of the common data set will encourage both animal and plant breeders to participate in the analysis.

## Competing interests

The authors declare that they have no competing interests.

## Authors' contributions

CM wrote the paper. CM and MB analyzed and compared the papers on QTL detection of participants, interpreted results and analyzed additional data sets to generate background information on the simulations. AC simulated the common data set, provided background information on various aspects of this data set and made the figures in R. JB, MC and AC contributed to critically revising the paper.

## Supplementary Material

Additional file 1
