# Supplementary material for: Comparison of analyses of the QTLMAS XIII common dataset. II: QTL analysis
Source: BMC Proc. 2010 Mar 31;4(Suppl 1):S2. doi: 10.1186/1753-6561-4-s1-s2 (PMC2857844; doi:10.1186/1753-6561-4-s1-s2)

## Additional file 1

### Analysis of the common and expanded data sets with FlexQTL

The common data set and the three expanded data sets were also analyzed with FlexQTL software [S1, S2] to evaluate the power to detect QTLs for the three parameters after estimating them from the time course data and to find out whether the range and intervals of the time points was limiting the success of QTL analysis. The FlexQTL software employs Markov Chain Monte Carlo (e.g., [S3]) simulation to perform a Bayesian analysis (e.g., [S4]) to map multiple QTL simultaneously. Each of the Markov chains resulted into 2000 samples with a thinning of 50 between consecutive samples. The prior distribution for the number of QTL was a Poisson distribution with mean equal to 5. The computation time ranged from 12 to 24 hours depending on the number of QTL that were fitted to the genetic model.

### Results

The results from the analyses by FlexQTL software show that if the correct logistic model is used to estimate parameters of the growth curve from the five time points of the common data set, all six QTLs for the asymptote can be found, five QTLs for the scaling factor, and maybe one for the inflection point. For the inflection point strong improvements in QTL detection are obtained if a larger time range is available and if the number of time intervals is increased (Figure 3, Appendix). The posterior probability of a QTL on chromosome 1 is increased considerably and evidence for QTLs on chromosomes 3, 4 and 5 is also found. There seemed some bias in the estimated positions of QTLs on chromosomes 4 and 5.

## References Additional file 1

S1. Bink MCAM., Boer MP, ter Braak CJF, Jansen J, Voorrips RE, van de Weg WE: **Bayesian analysis of complex traits in pedigreed plant populations.** *Euphytica* 2008, 161:85–96.

S2. Bink M, van Eeuwijk F: **A Bayesian QTL linkage analysis of the common dataset from the 12th QTLMAS workshop.** *BMC Proc* 2009, 3:S4.

S3. Gilks WR, Richardson S, Spiegelhalter DJ: **Markov chain Monte Carlo in practice**. London [etc.]: Chapman & Hall; 1996.

S4. Gelman A, Carlin JB, Stern HS, Rubin DB: **Bayesian data analysis**, 1st edition edn. London [etc.]: Chapman & Hall; 1995

**Figure S1, additional file 1**

QTL mapping results from analyses by FlexQTL software for $\phi_2$ (inflection point) on different data sets (W1-4) from which $\phi_2$ was estimated.

W1. Common data set: time frame of 0-530, time intervals of about 130, number of time points 5. W2. time frame of 0-1010, time intervals of about 130, number of time points 9. W3. time frame of 0-530, time intervals of 20, number of time points 27. W4. time frame of 0-1010, time intervals of 20, number of time points 51.

**Figure S1 Additional file1**

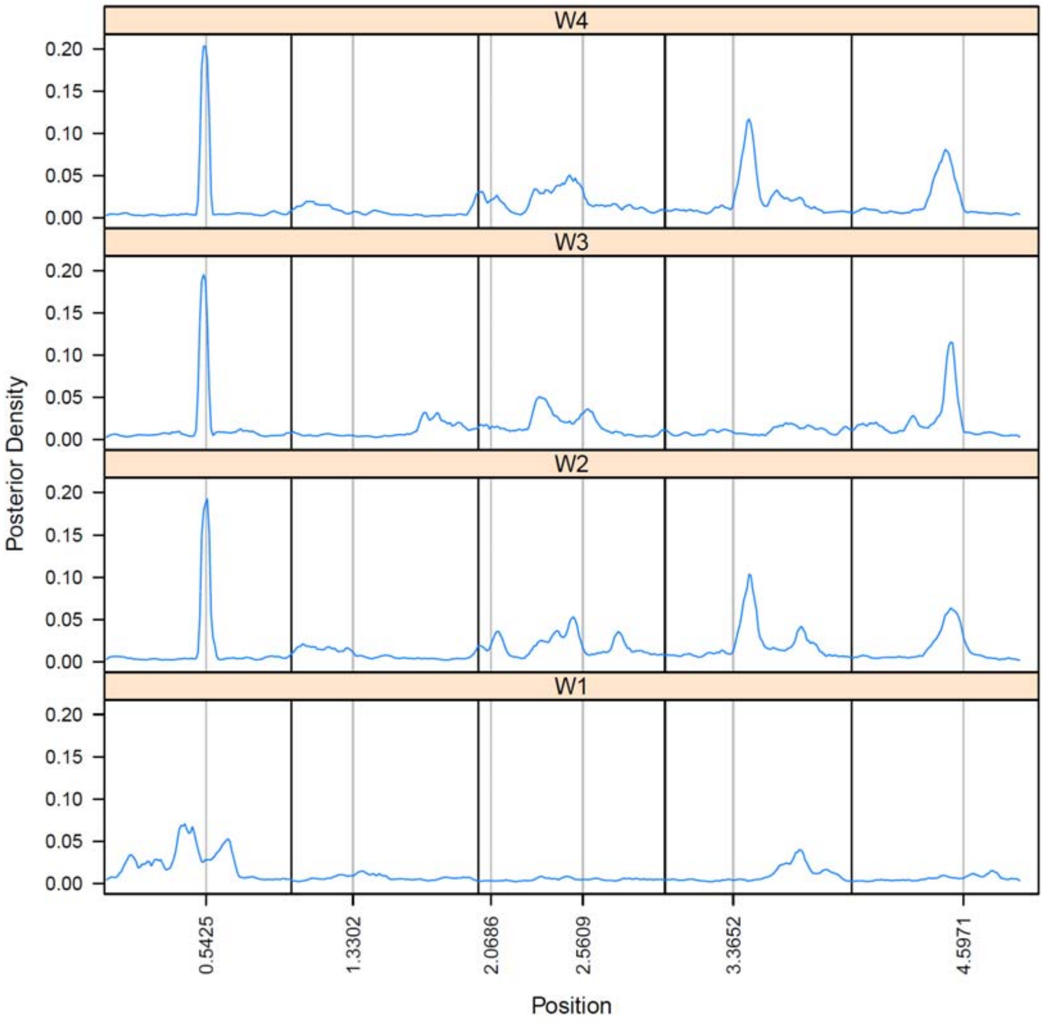

Supplement: Additional file 1 [file 1753-6561-4-S1-S2-S1.pdf]
